# Role of Multiple Comorbidities and Therapies in Conditioning the Clinical Severity of DRESS: A Mono-Center Retrospective Study of 25 Cases

**DOI:** 10.3390/ijms22137072

**Published:** 2021-06-30

**Authors:** Andrea Toniato, Chiara Gamba, Jan Walter Schroeder, Valeria Fabbri, Scarlett Valeria Bernal Ortiz, Linda Borgonovo, Marta Piantanida, Joseph Scibilia, Luca Balossi, Eleonora Brusamolino, Emanuela Bonoldi, Valentina Caputo, Michele Nichelatti, Elide Anna Pastorello

**Affiliations:** 1 Unit of Allergology and Immunology, ASST Grande Ospedale Metropolitano Niguarda, 20162 Milan, Italy; chiara.gamba@ospedaleniguarda.it (C.G.); janwaltervolk.schroeder@ospedaleniguarda.it (J.W.S.); valeria.fabbri@ospedaleniguarda.it (V.F.); Scarlett.Valeria.BernalOrtiz@ospedaleniguarda.it (S.V.B.O.); linda.borgonovo@ospedaleniguarda.it (L.B.); marta.piantanida@ospedaleniguarda.it (M.P.); giuseppe.scibilia@ospedaleniguarda.it (J.S.); lucagiuseppe.balossi@ospedaleniguarda.it (L.B.); eleonora.brusamolino@ospedaleniguarda.it (E.B.); elideanna.pastorello@ospedaleniguarda.it (E.A.P.); 2 Unit of Pathological Anatomy and Cytogenetics, ASST Grande Ospedale Metropolitano Niguarda, 20162 Milan, Italy; emanuela.bonoldi@ospedaleniguarda.it (E.B.); valentina.caputo@ospedaleniguarda.it (V.C.); 3 Service of Biostatistics, ASST Grande Ospedale Metropolitano Niguarda, 20162 Milan, Italy; michele.nichelatti@ospedaleniguarda.it

**Keywords:** DRESS syndrome, drug reaction with eosinophilia and systemic symptoms, drug-induced hypersensitivity syndrome, SCAR, severity, risk factors, comorbidities

## Abstract

DRESS/DiHS is a complex and potentially fatal drug reaction. Little is known about risk factors and elements that can help to identify patients with a severe reaction early. The aim of the study was to investigate those factors favoring the disease and its severity by analyzing the clinical conditions and therapies preceding the reaction. We conducted a retrospective analysis on patients admitted to our center between 2010 and 2020 who were discharged with a diagnosis of DRESS. We used the RegiSCAR diagnostic criteria. We defined the severity of DRESS using the criteria of Mizukawa et al. We included 25 patients (15 females) with a median age of 66 years. Skin involvement, eosinophilia, and liver injury were the most important aspects. Allopurinol was found to be the most involved drug. Reaction severity was significantly associated with the number of daily medications (*p* = 0.0067) and an age of at least 68 years (*p* = 0.013). In addition, 75% of severe cases had at least three comorbidities in history, and most of the severe cases were female. In our study the advanced age, the high number of comorbidities and home therapies, and the inflammatory state were found to be predisposing elements to the development of the disease and its severity.

## 1. Introduction

DRESS/DiHS is a complex and potentially fatal drug reaction that includes skin rash, eosinophilia, atypical lymphocytosis, lymphadenopathy, fever, and systemic organ involvement [1].

Its incidence is about 10 cases per 1 million inhabitants [1]. The mortality is around 10% in hospitalized patients [2]. The drugs mainly implicated are allopurinol and anticonvulsants, followed by antibiotics and proton pump inhibitors [3]. The precise pathogenesis of DRESS remains unknown. The immune mechanism is a IVb cell-mediated reaction, in which activated Th2 cells act by recruiting eosinophils. In addition, an important role is played by genetic predisposition conferred by particular polymorphisms in HLA genes with respect to specific drugs, like HLA-B*58:01 for allopurinol, HLA-A*31:01 for carbamazepine, and HLA-B*57:01 for abacavir [4]. Generally, data suggest the involvement of polymorphisms in genes encoding metabolic enzymes, such as CYP450 and N-acetyltransferase [5]. Moreover, other mechanisms involve altered drug detoxification or sequential reactivation of herpesviruses, such as human herpesvirus type 6 (HHV-6) and cytomegalovirus (CMV), and the subsequent antiviral immune response, which all appear to be associated with the systemic manifestations of DRESS [6].

The onset of DRESS occurs within the first two months after the introduction of the culprit drug and is characterized by prodromal symptoms like fever, pharyngodynia, and lymphadenopathy. The reaction usually lasts more than 15 days with several flare-ups [4]. Diagnosis is often difficult. Several diagnostic criteria have been developed, in particular the score of the RegiSCAR group [7] and the score of the J-SCAR [8], differing only for the reactivation of HHV-6, a criterion not included in the former.

Discontinuation of the culprit drug is sometimes sufficient to achieve resolution of the disease. To define the culprit drug, it is important to consider all drugs introduced in therapy in the 3–8 weeks preceding the onset of symptoms, but this can be complicated in the case of multiple therapies or a long latency between the start of therapy and the onset of the reaction.

Although in the process of being defined, the gold standard therapy is high doses of steroids for a prolonged period with slow decalage, otherwise there is a long-term risk of autoimmune disease [9]. Antiviral therapies are added in case of documented viral reactivation [1].

Problems remain open with respect to both the identification of the risk factors predisposing to DRESS and the recognition of the clinical elements that can identify early patients with a severe clinical picture who require a more aggressive and lasting treatment. The outcome of DRESS is often unpredictable, although a worse prognosis seems to be associated with the presence of severe liver damage, atypical lymphocytes, and viral reactivation [1]. In this regard, Mizukawa et al. proposed a score to determine the severity of the reaction and try to predict the risk of the occurrence of CMV reactivation in addition to the risk of complications [10].

Therefore, in this our work, we set out to investigate those factors favoring the disease and its severity. In this regard, we studied a group of patients with DRESS in whom the diagnosis was made strictly according to the current RegiSCAR criteria, specifying the probability of certainty. The aim of the study was to investigate the clinical situations preceding the reaction and the general context of pharmacological intake in which DRESS occurred. This was done with the aim of correlating the various parameters with each other to arrive at a definition of the criteria for the severity of the clinical picture.

## 2. Results

### 2.1. Patients Characteristics

As shown in Table 1, we included in our study 25 patients (15 females). The median age was 66 years (mean 62.04) with a slightly higher median values in females than in males (66 vs. 58.5 years). We also found a slight predominance of females in our population (male/female ratio: 0.66).

### 2.2. Clinical Results

Nine patients were defined as “definite cases” (36%), nine as “probable” (36%), and seven as “possible” (28%). The reasons for the uncertainty were derived not so much from the lack of key aspects for DRESS such as skin rash, eosinophilia, and organ involvement, but especially from the impossibility to exclude all those conditions that enter in differential diagnosis with DRESS according to RegiSCAR criteria for the lack of the results (see Table 2) [11,12].

As shown in Table 1, skin involvement was observed in 100% of cases, whereas a significant facial edema and mucosal involvement were rarer. Skin histology, performed in seven patients, reported pictures that excluded other diagnoses and were suggestive of DRESS, as the most reported alterations were represented by spongiosis, lymphocyte, and peri-vascular inflammatory infiltrate and apoptosis phenomena.

Hepatic involvement, atypical lymphocytosis, and hypereosinophilia were found to be other major features of the reaction (Table 1). An eosinophil elevation ≥ 700/μL was found in 84% of cases, of which 57% presented with a value ≥ 1500/μL. In four cases, no eosinophilia was found; in three of these, (3/25, 12%) this was probably in relation to the steroid therapy that was already started and in one case due to lack of data.

### 2.3. Other Laboratory Findings

**Figure 1:**
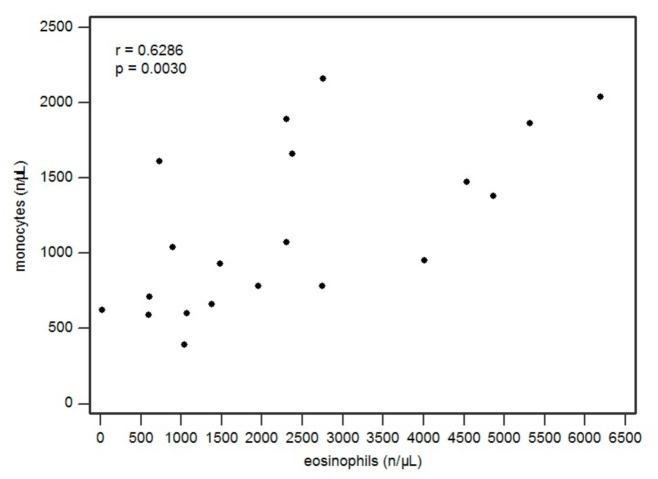
Scatterplot regarding correlation between eosinophil and monocyte values (in cells/μL).

Hematological alterations were found in all patients, as shown in Table 3. Monocytosis was found in 42% of cases (8/19). We found a correlation between monocyte and eosinophil levels (Pearsons r = 0.6286, *p* = 0.0030) (see Figure 1).

We detected an increase in C-reactive protein (CRP) in 19 out of 25 cases (76%) and an increase in erythrocyte sedimentation rate (ESR) in 5 out of 7 available cases; hyperferritinemia was highlighted in 7 of the 8 cases evaluated.

Viral reactivation of HHV-6 was documented in 16 of 17 cases by elevation of a specific IgG titer. CMV reactivation was found in four out of six cases by viral genome detection on peripheral blood.

Genomic typing of HLA was performed in 8 of 25 patients (Table 3).

### 2.4. Culprit Drugs

A culprit drug was identified and defined as certain in 19 of 25 patients (76%), as shown in Table 3. For the remaining six cases, it was not possible to identify the responsible agent because of a poly-administration of drugs, each characterized by a similar degree of causality.

### 2.5. Severity of the Reaction

According to Mizukawa severity score [10], the reaction was defined as moderate in 11 patients (44%) and severe in 13 (52%). For one patient, data were insufficient to define the severity.

**Figure 2:**
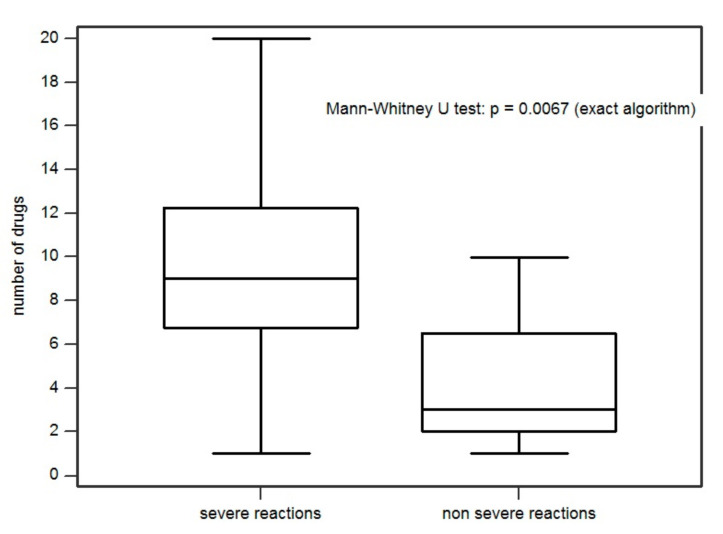
Association between reaction severity and number of medication in home therapy.

**Figure 3:**
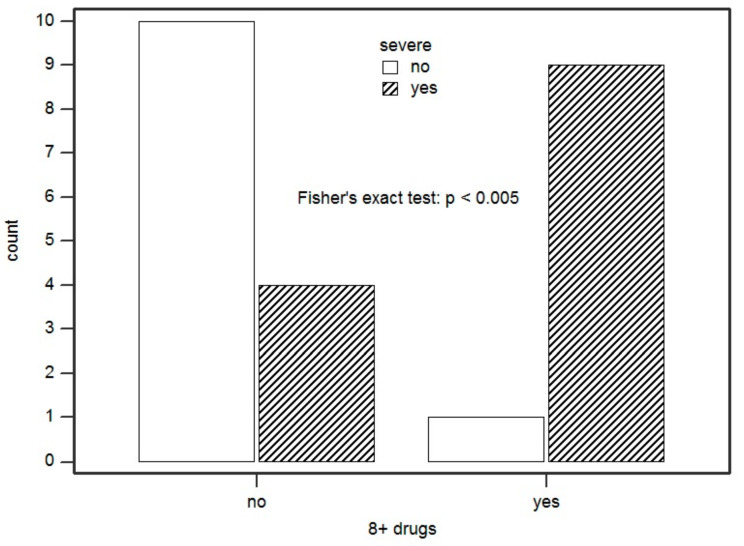
Association between having eight or more medications on therapy and developing a severe reaction. Count = number of patients.

The severity of the reaction was significantly associated with the number of medications taken daily by the patients (*p* = 0.0067). This means that there was an 81.8% probability that a patient with a severe reaction (SR) had a greater number of medications in therapy than a patient with a moderate reaction (Figure 2). We found that the number of medications in therapy prior to DRESS conditioned the severity of the reaction. Specifically, we set a significant cut-off at 8 drugs/day. Thus, we showed that every single additional drug in therapy increases the odds of having a SR by 41% (*p* = 0.016). In addition, having eight or more medications on therapy compared with having seven therapies or less increased the odds of developing a SR by 2050% (*p* = 0.010) (Figure 3).

**Figure 4:**
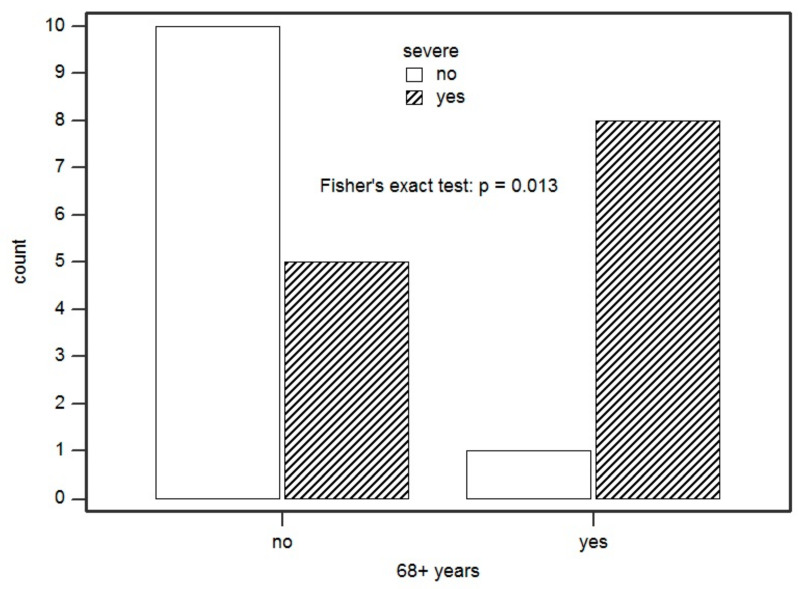
Association between being ≥68 years of age and presenting with a severe reaction. Count = number of patients.

Regarding demographic data, we found that 69.2% of subjects with a SR were female. It was also found that being female, compared to male, increased the odds of being severe by 91% (*p* = 0.05). The age of 68 years was found to be the cut-off that best distinguished our case series. Comparing it to reaction severity, being at least 68 years of age increased the odds of having a SR by 1600% compared to those with a lower age (*p* = 0.013) (Figure 4). In addition, it was found that 80% of those with eight or more medications on therapy were at least 68 years old (*p* = 0.002), and that all female subjects aged 68 years or more had a SR, with an increase of the odds of having a SR by 684% (*p* = 0.041).

It was also noted that all four lymphocytopenic patients presented with a severe grade reaction. In addition, these had an age of 68 years or older (*p* = 0.020) and were taking at least 8 medications/day (*p* = 0.012).

### 2.6. Patient Comorbidities and Chronic Therapies

**Figure 5:**
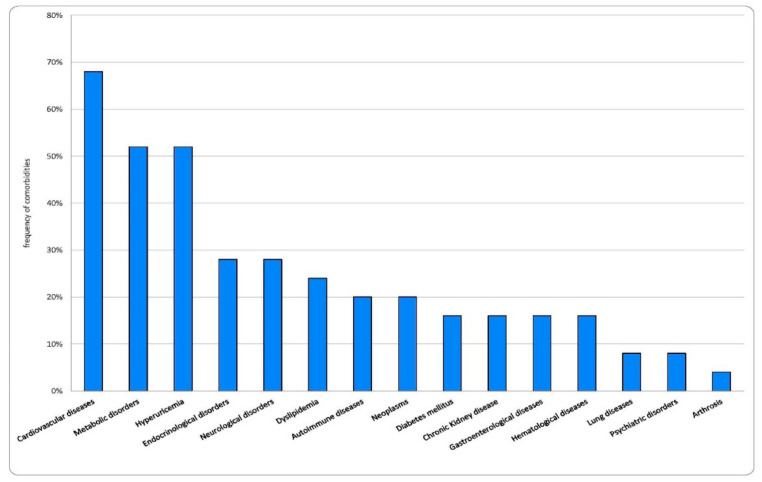
Frequency (%) of comorbidities found in our study population.

Comorbidities were found in 22 of 25 patients (88%). In total, 15 patients out of 25 (60%) had three or more comorbidities, 7 patients (28%) had two comorbidities and only 3 patients (12%) had one disease condition. Frequencies of comorbidities are shown in Figure 5. The most frequent comorbidities were cardiovascular disease (CVD), in particular hypertension (52%), followed by metabolic disorders such as hyperuricemia, dyslipidemia, and diabetes mellitus (DM). We compared the comorbidities found in our study with those described in other case reports in the literature (see Table 4) [11,13,14,15,16,17,18]. This analysis shows that hypertension, metabolic disorders, neurological diseases, and infectious diseases represent the most frequent comorbidities in the studies considered.

Regarding the medications taken daily by our patients, the average was 6.76 medications/day (SD 4.91). Notably, we found a number of drugs in therapy ≥ 4 drugs/day in 64% of cases (16/25), a number of 2–3 drugs/day in 24% of cases (6/25) and only 1 drug/day (allopurinol) in home therapy in 3/25 patients (12%).

### 2.7. Clinical Course

Complications were found in 28% of cases (7/25), as shown in Table 5. Two deaths were detected during the post-discharge follow-up: one at three months after admission for hemorrhagic stroke and the other after two years for glioblastoma. No autoimmune sequelae emerged during the follow-up period at this time.

Relapses were observed in 10 out of 21 cases (47.6%). In these cases, it was necessary to increase the steroid dosage and prolong the decalage.

All patients were treated with systemic steroid at a dosage of 1 mg/kg/day prednisolone or equivalent. Information regarding steroid therapy is highlighted in Table 1.

## 3. Discussion

In this study, we described a case history of subjects in whom DRESS was diagnosed, trying to highlight the most relevant aspects that, although generally conforming to known case histories, showed original aspects [11,13,14,19,20].

We must highlight that all patients had a skin rash, 84% of cases presented an increase in eosinophils and another 84% of subjects present an internal organ involvement, with a clear preponderance for hepatic involvement (54.1%), while there was renal involvement only in 37.5% of cases. Regarding hematological aspects, we found a lymphocytosis in the majority of cases. Although in some cases lymphopenia was found, the latter was described only in patients with SRs. This finding could be related to the immunosuppression typical of DRESS, which is also potentially related to herpesvirus reactivation [21]. In our study, we observed a marked increase in monocytes, which were significantly correlated with eosinophils (*p* = 0.0030). The phenotype of these monocytes is unknown, but this finding seems of particular interest also in light of studies in the literature, such as that of Kang et al., which showed that monocytes of “non-classical-type” in DRESS are cells that are able to act as pharmacological antigen-presenting cells once activated by inflammatory cytokines and, at the same time, are endowed with a pronounced dermotropism [22], being precisely the cells involved in the skin manifestation.

In line with the data from the above literature, we found leukocytosis in 80% of cases and neutrophilia in 66.6% of our cases, similar to the frequency of atypical lymphocytosis (64% of patients) [11] and lymphocytosis [14]. In contrast, lymphadenopathy was found in 24% of our cases, a lower rate than that described in Kardaun et al. [11] and Avancini et al. [19].

Our results show the important role of allopurinol, which was the causative drug in 48% of cases. A correlation with the presence of the HLA-B*58:01 was also confirmed in the cases studied, as is known in the literature [4]. In this regard, it is interesting to note that 20% of our population was represented by Asian patients, a significant percentage compared to the other Asia population in Italy. As known in the literature, this finding may be related to pharmacogenetic aspects, as Asian subjects have a higher frequency of the allele HLA-B*58:01, which is associated with an increased risk of DRESS caused by allopurinol. This aspect was also confirmed by our study as all Asian patients presented with DRESS caused by allopurinol and were found to be carriers of the allele HLA-B*58:01. After allopurinol, aromatic anticonvulsant drugs were found to be the most involved, as described in the literature [1,20]. In 24% of the cases in our case series, it was not possible to trace the responsible drug, a finding comparable to the literature according to which in 10–20% of cases diagnosed as DRESS, the relationship with a precise causal drug cannot be established [23].

Reactivation of HHV-6, when evaluated, was frequent and evidenced by a significant increase in specific IgG. CMV reactivation was found in 66.6% of the cases in which it was evaluated, all of which were represented by patients who presented a SR. In fact, CMV reactivation is considered an index of clinical severity, so much so that it was analyzed by Mizukawa et al., who proposed a score composed of clinical and biological parameters to establish the severity of the reaction and thus predict the risk of viral reactivation, which is often associated with longer clinical courses and the onset of complications [10]. Using this score, in our case series, we tried to analyze the severity of the reaction, which was found to be not only related to the immune-mediated mechanism, but also, and perhaps above all, to the patient’s basic clinical background. First of all, the age of the subject was found to be an aspect significantly correlated with the severity of the reaction with a cut-off of 68 years of age that was found to be suitable for discriminating between severe and non-SR in our population (*p* = 0.013). In addition, the female sex is known to be the most affected by hypersensitivity reactions to drugs and, in our case series, was found to be significantly correlated with the severity of the reaction in subjects older than 68 years (*p* = 0.041). Analyzing the pathological history preceding the onset of the reaction, we observed how the poly-pharmacological intake was the most predisposing element to the occurrence of a particularly severe DRESS syndrome. In particular, it is interesting to note that we identified a number of daily drugs considered as a cut-off discriminating between a SR or not (8 drugs, *p* = 0.0067). Finally, the presence of multiple underlying chronic pathologies was also an important element, as well as having at least three documented pathologies in the history, which was found to be present in 75% of subjects with SR.

With regard to the comorbidities found in our study, CVD (especially hypertension) and metabolic diseases (especially hyperuricemia, dyslipidemia, and DM) were the most frequently described. These findings are in agreement with what has been observed in other studies and show that most of the comorbidities are not linked to allergic or traditionally eosinophilic aspects. On the contrary, as known in the literature, these comorbidities are associated with an inflammatory state and an altered drug detoxification metabolism [24,25]. These events can promote the release of inflammatory mediators (such as interleukins and alarmins), the altered integrity of cell membranes, especially epithelial ones, and finally, mechanisms of drug toxicity or hypersensitivity.

All these elements, such as the involvement of drugs with particular characteristics, the pre-existence of multiple diseases, polypharmacy, age, and female sex, would seem to favor this rare and particular reaction in which there are two key aspects: the increase of eosinophils and the frequent hepatic involvement. It is conceivable that age and severe pre-existing chronic diseases have influenced a decreased ability to detoxify drugs with an increase in circulating reactive metabolites, as also reported in the literature for cases of DRESS associated with anticonvulsant drugs [26]. These reactive compounds, harmful for cell membranes, could determine a cellular necrosis with consequent release of cellular components able to act as danger signals. In some cases, these would be real alarmins, among which it has been hypothesized that a particular role is played by high-mobility group box 1 (HMGB-1), an alarmin that has been found in high levels both on blood and skin in patients affected by DRESS [27]. In addition to this, HMGB-1 is able to stimulate the synthesis of eosinophils, whose increase would not only be associated to Th2 stimulation, but also to the activation of innate immunity. As known, this powerful alarmin also attracts neutrophils with high inflammatory potential and, above all, monocytes that would be able to infiltrate the skin under the stimulus of HMGB-1 [28,29,30].

From this analysis comes the realization that in individuals with multiple diseases, taking numerous medications, and at an advanced age, the use of some medications may be more dangerous than for other patients and, therefore, the risk of DRESS should be considered in their prescription. This is particularly true for some usually implicated drugs such as allopurinol and anticonvulsant drugs, which act not only in an HLA genotype-dependent manner, but also act independently, as seen in our study, involving other mechanisms, for example, related to stimulation of innate immunity or damage on cell membranes. In this regard, Nakajima et al. demonstrated that allopurinol is also able to induce an innate response at the level of dendritic cells, acting directly as danger signals [31], a finding in line with other studies in the literature [32,33,34], and with the above considerations. This draws attention to the “chemical stress” linked to many chemicals, including drugs, the danger signals related to it and the ability of chemicals to modulate and induce the immune response [34].

## 4. Materials and Methods

### 4.1. Patients

We conducted a retrospective analysis on patients admitted to the ASST GOM Niguarda in Milan between 2010 and 2020 and discharged with a diagnosis of DRESS made by the allergist of our team who was called in to consult for patients with skin rash.

Demographic data and data relating to current and previous clinical history were collected for each patient. With regards to the current history, we investigated the conditions for which the drug was prescribed, the initial symptoms, the full clinical picture, and the evolution. Particular attention was given to each patient’s daily medication intake, focusing on drugs taken because of underlying conditions. With regard to the previous history, we collected the relevant pathologies, focusing on the presence of comorbidities, defined as pathologies already diagnosed before the drug reaction and evaluated when they were clinically well defined and documented. We focused, in particular, on the presence of CVD, renal and hepatic diseases, metabolic and endocrinological disorders, neoplasms, autoimmune diseases, neurological disorders, and pulmonary diseases. Finally, we analyzed the allergy history and the presence of previous drug reactions.

### 4.2. Methods

#### 4.2.1. Diagnosis

We used the diagnostic criteria proposed by RegiSCAR, as shown in Table 2 [11,12]. Patients with a score > 2 were included in the study.

Laboratory investigations included CRP positivity (>0.5 mg/dL), hyperferritinemia (>400 ng/mL), and ESR elevation (>10 mm/1 h). Leukocytosis was defined as values >10,000 cells/μL, neutrophilia as values >7000 cells/μL, and monocytosis as values >1200 cells/μL. The normal range considered for lymphocytes was 800–5000 cells/μL, while that of platelets was 140,000–400,000 cells/μL.

Among the viral investigations, we searched for specific IgG and IgM for HHV-6 and CMV and the viral genome by polymerase chain reaction (PCR) for HHV-6 (lower limit of the linearity range of 250 gEq/mL) and for CMV (lower limit of the linearity equal to 100 copies/mL).

We analyzed, when possible, the HLA genomic typing investigations carried out using the PCR-SSO method.

For each patient, we analyzed the drugs involved trying to identify a causal relationship. This was assessed based on the six categories proposed by the criteria of the Uppsala Monitoring Center of the World Health Organization (WHO-UMC). In this way, the relationship is defined as certain, probable, possible, improbable, conditional/unclassified, and unassailable/unclassifiable [35].

The latency period was defined as the time elapsed between the start of the responsible drug, where certain, and the index day, represented by the day of onset of the skin symptoms suggestive for DRESS.

The severity of the reaction was assessed by the score presented by Mizukawa et al. [10] that defines the severity of DRESS in early and late stages. The parameters used are defined as fixed or variable, as shown in Table 6. Therefore, we looked for a relationship between severity of the reaction and the number of drugs or comorbidities.

Moreover, the number of drugs in therapy and the prevalence of comorbidities found were compared with official Italian epidemiological case reports analyzed by ISTAT data in 2018 [36] and ministerial data from the Superior Institute of Health [37]. We focused our attention, in particular, on the elderly population, dwelling on the pathological conditions considered to cause clinical complexity and severity, such as CVD, neoplasms, pulmonary diseases, DM, liver diseases, chronic kidney disease (CKD), arthrosis, allergic diseases, neurological diseases, and gastric ulcer.

#### 4.2.2. Clinical Course

Complications were considered the pathological conditions that occurred during the disease. Among them, we considered CVD, such as heart failure, heart attack, or stroke, as well as sepsis, gastrointestinal bleeding, autoimmune or hematological diseases, and death from DRESS.

Relapse was considered the resumption of skin symptoms, systemic damage, and/or the rise of eosinophils. For autoimmune sequelae, we considered the onset of autoimmune conditions during the one-year follow-up after discharge.

We analyzed steroid therapy by attempting to establish, where available from clinical documentation, the dosage, the duration of initial high-dose therapy (1 mg/kg/day), and the overall duration of therapy taking into account decalage. We then defined treatment latency as the period elapsed between the index day (see above) and the day of steroid initiation.

#### 4.2.3. Statistical Analysis

The data obtained was collected in a database using the Microsoft (MS) Excel data analysis program. Age was expressed as median and standard deviation. Data were expressed as absolute numbers and/or as percentages relative to the sample whose information was available. We relied on the Mann–Whitney exact algorithm U-test and Fisher’s exact test to determine differences between groups with regard to continuous and binary variables, respectively. To assess whether an independent variable had a statistically significant relationship with the dependent variable, we relied on Wald’s test with an algorithm for rare events. Correlation was assessed using Pearson’s correlation coefficient (r). All statistical analyses were performed using Stata/SE 16.1 software (The StataCorp LLC, College Station, TX, USA). Relationships with a *p*-value <0.05 were considered statistically significant.

## 5. Conclusions

In conclusion, DRESS is a severe drug reaction that can potentially occur with all drugs, not just those most frequently involved. For this reason, the risk of DRESS should be considered at the time of prescribing, particularly in the case of patients with particular characteristics. Indeed, although there is a limit to the number of patients and our analysis needs further investigation, in our study, advanced age, a high number of comorbidities, a high number of home therapies, and an inflammatory state were found to be predisposing elements in the development of the disease and its severity. This leads us to assume that the disease is linked to an altered drug metabolism that, through the production of reactive metabolites, determines the activation of innate immunity leading to the induction of a hypersensitivity mechanism.

## Figures and Tables

**Table 1:** Characteristics of 25 patients with DRESS syndrome.

Characteristics	Number
**Median age, years**	66
**Female (n, %)**	15 (60%)
**Ethnic group**	
Caucasian	19 (76%)
Asian	5 (20%)
African	1 (4%)
**Community cases (n, %)**	19 (76%)
**Duration of hospitalization (median ± SD), days**	15 ± 10.8
**Skin (n, %)**	25/25 (100%)
>50% BSA	25/25 (100%)
Rash suggestive of DRESS *	25/25 (100%)
Mucosal involvement	8/25 (32%)
Facial edema	9/25 (36%)
**Lymphadenopathy (n, %)**	6/25 (24%)
**Fever ≥38.5 °C (n, %)**	13/25 (52%)
**Atypical lymphocytosis (n, %)**	16/25 (64%)
**Eosinophilia**	
≥700/μL (n, %)	21/25 (84%)
≥1500/μL (n, %)	12/25 (48%)
**Hematological abnormalities (n, %)**	25/25 (100%)
Leukocytosis	20/25 (80%)
Neutrophilia	16/24 (66%)
Monocytosis	8/19 (42%)
Lymphocytosis	6/24 (25%)
Lymphocytopenia	4/24 (16%)
Trombocytosis	3/24 (12.5%)
Trombocytopenia	7/24 (29.1%)
**Internal organ involvement (n, %)**	21 (84%)
Liver (%)	54.1%
Kidney (%)	37,5%
Heart (%)	12.5%
Lung (%)	8%
**Number of organs involved**	
1 organ (%)	63%
2 organs (%)	17%
≥3 organs (%)	4%
**Culprit drugs**	
Certain culprit drugs (n, %)	19 (76%)
Latency ** (median ± SD), days	21 ± 12.7
**Steroid treatment**	
Overall duration (mean SD), days	135.8 ± 226.6
Treatment start latency (mean SD), days	14.75 ± 17.5
1 week (at least) at 1 mg/kg/day *** (n, %)	12 (48%)
2 weeks at 1 mg/kg/day *** (n, %)	4 (16%)
3 weeks at 1 mg/kg/day *** (n, %)	1 (4%)

SD: standard deviation; BSA: body surface area. * as described in Kardaun [11,12]. ** latency between initiation of therapy and the onset of the reaction (for certain culprit drugs). *** 1 mg/kg/day prednisolone or equivalent.

**Table 2:** RegiSCAR score system. Table modified from Kardaun et al. [11,12].

Score	−1	0	1	2	Min	Max
**Fever ≥ 38.5 °C**	No/U	Yes			−1	0
**Lymphadenopathy**		No/U	Yes		0	1
**Eosinophilia** Eosinophils Eosinophils, if leukocytes < 4000			700–1499/μL 10–19.9%	≥1500/μL ≥20%	0	2
**Atypical lymphocytes**		No/U	Yes		0	1
**Skin involvement** Rash extent (>50% BSA) Rash suggesting DRESS Biopsy suggesting DRESS	No No	No/U U Yes/U	Yes Yes		−2	2
**Organ involvement** * Liver, kidney, lung, muscle/heart, pancreas, other organ (s)		No/U	Yes		0	2
**Resolution ≥ 15 days**	No/U	Yes			−1	0
**Evaluation other potential causes** ANA, blood culture, serology for HVA/HBV/HCV, C./M. Pneumoniae, other serology/PCR if none are positive and ≥3 above negative			Yes		0	1
**TOTAL SCORE**					−4	9

U = unknown/unclassifiable; BSA = body surface area; PCR = polymerase chain reaction; C./M. = Chlamydia/Mycoplasma. * after exclusion of other explanations: 1 = 1 organ; 2 = ≥2 organs. Final score: <2 = no case; 2–3 = possible case; 4–5 = probable case; >5 = definite case.

**Table 3:** Culprit or suspected drugs and HLA genotyping.

Cases	Certain Culprit Drug	Drug(s) Involved	HLA
1	Yes	Allopurinol	HLA-B*58:01
2	Yes	Allopurinol	U
3	No	Allopurinol, ceftriaxone	U
4	Yes	Allopurinol	HLA-B*58:01
5	Yes	Allopurinol	U
6	Yes	Phenytoin	U
7	Yes	Allopurinol	U
8	Yes	Allopurinol	HLA-B*51:06 HLA-B*15:02
9	Yes	Carbamazepine	HLA-B*35:08 HLB-B*39:01
10	No	Allopurinol, cefpodoxime	U
11	Yes	Carbamazepine	U
12	Yes	Thiamazole	U
13	Yes	Allopurinol	U
14	Yes	Allopurinol	U
15	No	Carbamazepine, Lamotrigine, and Gabapentin	U
16	Yes	Allopurinol	HLA-B*58:01
17	Yes	Carbamazepine	HLA-B*18:01 HLA-B*40:01
18	Yes	Ticlopidine	U
19	Yes	Ciprofloxacin	U
20	Yes	Allopurinol	HLA-B*58:01
21	Yes	Allopurinol	U
22	No	Meropenem, Vancomycin, and Dalteparin	U
23	No	Allopurinol, Meropenem, Vancomycin, Bactrim (TMP/SMX), and Fluconazole	U
24	Yes	Allopurinol	HLA-B*58:01
25	No	Aripiprazole, Baclofen, Vortioxetine	U

TMP/SMX = Trimethoprim/Sulfamethoxazole.

**Table 4:** Comorbidities found in our study compared with other case series.

	Our Study	Wolfsong 2019	Kardaun 2013	Hirans. 2016	Sim 2019	Wang 2017	Ushigome 2012	Kim 2014
**N° patients**	25	69	117	52	123	104	34	48
**Age (mean/median)**	66 (median)	60 (median)	48 (median)	33 (median)	54.3	52 (mean)	55.5 (mean)	
**Hypertension**	13 (52%)	38 (55%)		13 (25%)	55 (45%)	22 (21.2%)		15 (31%)
**Hyperuricemia**	13 (52%)			8 (15.4%)		7 (6.7%)	4 (12%)	6 (12.5%)
**DM**	4 (16%)	12 (17%)	14 (12%)	8 (15.4%)	20 (16%)	10 (9.6%)		2 (4%)
**CKD/kidney diseases**	4 (16%)	8 (12%)	7 (6%)	4 (7.7%)	9 (7%)	4 (3.8%)		2 (4%)
**CAD**	3 (12%)	9 (13%)						
**CHF**		7 (10%)						3 (6%)
**Other CVD**	4 (16%)							
**Structural heart diseases**	6 (24%)							
**Arrhythmia**	4 (16%)						1 (3%)	
**Neoplasms**	5 (20%)	8 (12%)	6 (5.1%)		11 (9%)	2 (1.9%)	1 (3%)	2 (4%)
**Stroke**	1 (4%)						5 (15%)	
**Neurological diseases**	7 (28%)	13 (19%)	23 (20%)	12 (23.1%)		4 (3.8%)	11 (33%)	3 (6%)
**Autoimmune diseases**	5 (20%)	7 (10%)	10 (8.5%)			4 (3.8%)	3 (9%)	
**Lung diseases**	2 (8%)	10 (14%)			1 (1%)		2 (6%)	1 (2%)
**Psychiatric disorders**	2 (8%)	6 (9%)						4 (8%)
**Hematological diseases**	4 (16%)							1 (2%)
**Endocrinological diseases**	7 (28%)							
**Gastroenterological diseases**	4 (16%)		6 (5.1%)			7 (6.7%)	1 (3%)	
**Arthrosis**	1 (4%)							
**Dyslipidemia**	6 (24%)			9 (17.3%)				
**HIV**		3 (4%)	1 (1.3%)	15 (28.8%)				
**Tuberculosis**						4 (3.8%)		6 (12.5%)
**Recent infectious disease**			25 (22.9%)					
**Drug addiction**		7 (10%)						
**Allergic rhinitis**					1 (1%)			
**Atopic dermatitis**					1 (1%)			
**N°** **comorbidities** **1** **2** **≥3** **>4**	3 (12%) 7 (28%) 15 (60%)	31 (45%) * 29 (42%) # 9 (13%)					31 (91%) 2 (6%) 1 (3%)	

*: patients with 0–1 comorbidities; #: patients with 2–3 comorbidities. N° = number; DM = diabetes mellitus; CKD = chronic kidney disease; CAD = coronary artery disease; CHF = congestive heart failure; CVD = cardiovascular disease. References: Wolfsong et al. [13], Kardaun et al. [11]; Hiransuthikul et al. [14]; Sim et al. [15]; Wang et al. [16]; Ushigome et al. [17]; Kim et al. [18].

**Table 5:** Complications described in the case history.

N	Drugs Involved	Severity	RegiSCAR Score	Complications
1	Allopurinol, Cefpodoxime	Moderate	Probable	Hemolytic anemia and AITP
2	Allopurinol	Severe	Probable	Congestive heart failure
3	Allopurinol, Ceftriaxone	Moderate	Definite	Congestive heart failure
4	Allopurinol	Severe	Probable	Gastrointestinal bleeding
5	Allopurinol	Moderate	Definite	Hepato-renal decompensation
6	Allopurinol	Severe	Definite	Sepsis by Gemella
7	Thiamazole	Severe	Probable	Septic pneumonia turned out to be fatal

AITP = autoimmune thrombocytopenia.

**Table 6:** Severity score of DRESS reaction. Table modified from Mizukawa et al. [10].

	Values	Score
**Fixed parameters**		
Age (years)	≤40/41–74/≥75	−1/0/2
Duration of drug exposure after onset (days)	0–6/≥7	0/1
Atypical lymphocytes	NO/YES	0/1
**Variable parameters**		
Pulsed prednisone *	NO/YES	0/2
Skin involvement erythema (% BSA) erosion (% BSA)	<70/≥70/erythroderma <10/10–29/≥30	0/1/2 0/1/3
Fever ≥ 38.5 °C (days duration)	0 or 1/2–6/≥7	0/1/2
Appetite loss (≤70% of regular food intake), days	0–4/≥ 5	0/1
Renal dysfunction (creatinine), mg/dL	<1.0/1.0–2.0/≥2.1 or HD	0/1/3
Liver dysfunction (ALT), IU/L	<400/400-1000/>1000	0/1/2
C-reactive protein (mg/dL)	≤2/<2–<10/≥10–<15/≥15	−1/0/1/2

Each variable parameter can be determined at early (days 0–3 after the initial presentation) and later times (2–4 weeks after the initial presentation), and on an as-needed basis. ALT = alanine aminotransferases; BSA = body surface area; HD = hemodialysis. * intravenous methylprednisone use ≥500 mg/day for 3 days. Score = −1: mild reaction; 1–3 moderate reaction; ≥4: severe reaction.
